# Editorial: Innovative Therapeutic and Vaccine Approaches Against Respiratory Pathogens

**DOI:** 10.3389/fimmu.2019.02960

**Published:** 2019-12-17

**Authors:** Cynthia Calzas, Delphyne Descamps, Michel Chignard, Christophe Chevalier

**Affiliations:** ^1^VIM, INRAE, Université Paris-Saclay, Jouy-en-Josas, France; ^2^Sorbonne Université, UPMC Univ. Paris 06, INSERM, Centre de Recherche Saint-Antoine, Paris, France

**Keywords:** vaccine, therapeutics, innovative delivery system, adjuvant, drug repurposing, antimicrobial peptide, respiratory pathogen

As recently reported by the Pneumonia Etiology Research for Child Health (PERCH) study group (1), the diseases responding to the WHO's definition of pneumonia, including pneumonia of bacterial or viral origin but also acute and classical bronchiolitis, are the leading killers of children worldwide (2 million children each year). These infections are usually transitory and can be treated symptomatically in adults. However, complications can result in superinfections originating in many cases by an acute viral or bacterial upper respiratory tract infection followed by invasion of the lower respiratory tract by bacteria, notably in children and in elderly or immunocompromised individuals. The PERCH study estimated that 61% of pneumonia requiring hospitalization in children had a primary viral cause. Respiratory syncytial virus (RSV) arrives on top of a list that also includes rhinovirus, human metapneumovirus (hMPV), parainfluenza virus, and influenza A virus (IAV). Bacterial pathogens such as *Streptococcus pneumoniae, Haemophilus Influenzae*, and *Mycobacterium tuberculosis* are also represented in that list. The development of new prophylactic and therapeutic approaches to reduce the morbidity and the mortality associated with these infections is thus critically needed.

In this Research Topic, a series of original articles and reviews provide some insights on new molecular and cellular therapeutic targets or innovative vaccine strategies against respiratory pathogens.

Current licensed vaccines against IAV are mainly inactivated or live-attenuated viruses that provide only an incomplete protection, most notably for groups at risk. Furthermore, the intranasal route appears to be a promising strategy of inoculation to fight against IAV directly at the primary portal of virus entry compared to classical parenteral administration. Calzas and Chevalier review the development of innovative delivery/adjuvant systems used for intranasal instillation of inactivated influenza vaccines, including micro/nanosized particulate carriers such as lipid-based particles, virus-like particles (VLPs), and polymers associated or not with immunopotentiatory molecules including microorganism-derived toxins, TLR ligands, and cytokines. In their mini review, Al-Halifa et al. present an overview of the advantages and limitations of the use of nanoparticle-based vaccines i.e., polymeric, inorganic, and self-assembling protein nanoparticles (VLPs) against respiratory viruses. The development of these new vaccines highlights the recent advances in chemical and biological engineering which allow the controlled design of safe nanoparticles (in size, shape, and functionality) to enhance antigen presentation and strong immunogenicity. The capacity of these vaccines to trigger specific mucosal and systemic humoral and cellular responses against respiratory pathogens and their (cross)-protective potential are also explored in those two reviews. The efficacy of vaccines is also correlated with the matching between the circulating and the vaccine strain, notably for IAV or RSV which are subject to a constant antigenic drift. In their original research article, Bernasconi et al. describe the development of a broadly protective universal influenza vaccine based on porous nanoparticles of maltodextrin incorporating recombinant self-adjuvanted M2e (ectodomain of the matrix 2 protein of IAV which is highly conserved among IAV strains) and hemagglutinin. They demonstrate that the intranasal instillation of their vaccine enhances immune protection against live homologous or heterologous IAV infections and decreases the risk of virus transmission. The protection is mediated by specific mucosal and systemic humoral and cellular responses. The outcome of vaccination can also be impacted by the phenomenon known as the original antigenic sin mostly associated with pre-existing antibodies against close viral strains that might impair antibody formation against previously unseen strains. Nienen et al. elucidate the role of IAV-specific helper T cells upon vaccination with unexperienced IAV strains in a healthy adult human cohort. In this original article, authors reveal that the pre-existing cross-reactive memory T cells provides sufficient help to naive B cells specific to previously unseen IAV strains and their baseline quantity directly correlated with vaccination efficacy.

Another alternative against respiratory pathogens is the development of anti-viral approaches. The dynamics of evolution, emergence and resistance of respiratory pathogens such as IAV highlights the critical need to enlarge the therapeutic arsenal available. Pizzorno et al. summarize in their review the state-of-the-art of current antiviral options against IAV infection and focus on the recent advances of anti-IAV drug repurposing strategies. The development of new approaches based on the combined targeting of host cell and the viral components could constitute effective strategies to avoid the emergence of resistant IAV mutants as often observed with the use of conventional antivirals. Many strategies are exploited to achieve this goal and are well illustrated in the review with examples ranging from serendipitous observations to *in silico*-assisted repurposing. In the original article associated to this review, Pizzorno et al. present the implementation of drug repurposing by exploiting *in vivo* global transcriptomic signatures of IAV-infected patients to determine the potential of already marketed drugs with newly identified inhibitory properties against IAV. Among a list of promising candidates, they demonstrate that diltiazem, a calcium channel blocker used to treat hypertension, in combination with oseltamivir increases antiviral efficacy. In their work, Fusade-Boyer et al. identify Sephin1, an inhibitor of cellular phosphatase, as an antiviral molecule against RNA and DNA viruses, and notably RSV. Nyanguile reviews peptide-based antiviral strategies against RSV and IAV. Long-acting macrocyclic peptides targeting large protein-protein interactions could be used to target critical regions such as the IAV hemagglutinin stalk domain or the RSV fusion protein to impede virus fusion. Finally, Le Nouën et al. review synonymous recoding strategies used to attenuate RSV and IAV: deoptimization of codon or codon-pair usage, reduction of viral protein expression, increase of the content of immunomodulatory CpG and UpA RNA dinucleotides and substitution of codons limiting evolutionary potential of the virus by increasing the probability of insertion of non-sense codons. The accumulation of synonymous mutations inserted to obtain the deoptimized virus should reduce drastically the risk of reversion while preserving the integrity of viral antigens. Such deoptimized IAV and RSV viruses have been generated and their characterization as vaccine candidates is described in this review.

With the emergence and spread of drug-resistant strains of *M. tuberculosis*, there is an urgent need to develop alternative anti-tuberculosis strategies. The parenteral live attenuated Bacillus Calmette-Guérin (BCG) vaccine, widely used during the past decades, protects infants and children against severe extra pulmonary forms of tuberculosis. However, it is inconsistently efficient against the most common respiratory form of the disease (pulmonary tuberculosis), is known to cause adverse effects in immunocompromised individuals and does not prevent the establishment of latent persistent infection. In recent years, a better understanding of the immunopathogenesis of *M. tuberculosis* infection allowed the development of more efficient anti-tuberculosis strategies, including the generation of more refined subunit vaccines and host-directed therapies (HDTs).

A proposed effective vaccine strategy is to trigger mucosal and/or parenteral host immunity with selective recombinant *M. tuberculosis* antigens associated with suitable delivery/adjuvant systems. Hu et al. demonstrate that a prime with BCG vaccine followed by a boost with a novel intranasal Sendai virus vectored vaccine encoding *M. tuberculosis* immunodominant antigens enhance the generation of specific systemic and lung poly-functional CD4^+^ and/or CD8^+^ T cell responses in mice. The authors suggest the improved protection against *M. tuberculosis* infection subsequent to the prime-boost immunization regimen is associated with higher levels of recall IL-2-mediated lung CD4^+^ and CD8^+^ T cell responses and a higher frequency of central memory CD4^+^ T cells in the lung. Thakur et al. evaluate for the first time the immunogenicity of a multistage tuberculosis subunit vaccine combining early antigens and a latency-associated protein with liposome-based cationic adjuvant upon parenteral prime and intrapulmonary boost administration in mice. Stronger systemic and lung antigen-specific polyfunctional CD4^+^ T cells and IgA responses are elicited with this vaccination course in comparison with parenteral prime-boost vaccination. By using non-invasive tomography imaging, the authors gain information on the anatomical biodistribution and pharmacokinetics of the vaccine, which could help in the development of effective mucosal vaccines against pulmonary tuberculosis. Finally, based on structural and functional analyses of domains III and IV of *Staphylococcus aureus* immunomodulatory protein Sbi (Sbi-III-IV), Yang et al. rationally design an auto-adjuvanted fusion protein vaccine against *M. tuberculosis*. By harnessing the alternative complement pathway dysregulating function of Sbi-III-IV, the authors improve immune responses against a *M. tuberculosis* vaccine antigen administered via the parenteral route in mice through its coating with C3 breakdown fragments.

Soto et al. demonstrate that the use of the BCG vaccine as a vector for recombinant expression of heterologous antigens is an attractive vaccine approach against RSV and hMPV. Recombinant BCG vaccines expressing either the nucleoprotein of RSV or the phosphoprotein of hMPV induce a cellular immune response able to boost the humoral response against RSV or hMPV antigens beyond those encoded by the vaccines and prevent the disease caused by both pneumoviruses in mice. A pathological hallmark of tuberculosis is the formation of granulomas in the lung, which are organized immunological structures composed of various innate and adaptive immune cells containing the pathogen. However, granulomas can undergo complex structural changes resulting in tuberculosis progression and attractive HDTs against tuberculosis consist in targeting granulomas. Remot et al. review and discuss the role of neutrophils within the tuberculosis granuloma and the impact of the hypoxic environment encountered in the tuberculosis granuloma on key neutrophil-released mediators. The authors highlight the modulation of hypoxia-induced factors as an attractive innovative HDT against tuberculosis. Jones et al. also target neutrophils to fight against *Aspergillus fumigatus* by using bifunctional compounds combining moieties that bind to the surface of the pathogen and moieties that interact with chemoattractant receptors on human neutrophils. The authors show that these compounds enhance the activity of neutrophils against *Aspergillus fumigatus in vivo* and *in vitro*, using a zebrafish infection model and using neutrophils isolated from healthy humans and immunosuppressed patients, respectively.

Voβ et al. validate that the intranasal vaccination with lipoproteins is a new protective strategy against nasopharyngeal colonization by *S. pneumoniae*. Lipoproteins (PnrA, DacB, and MetQ) from pneumococcal serotypes, known to act as adhesins, are abundant at the surface of the pathogen, conserved, and highly immunogenic in mice. The knowledge of host-pathogen interactions and of the mechanisms of immune responses allows proposing innovative immunomodulatory strategies by targeting innate receptors, such as Toll-like receptors (TLRs), to selectively boost innate immunity and therapeutic treatment outcome. In an original research article, Matarazzo et al. demonstrate that the local delivery in the respiratory tract of flagellin, a natural agonist of TLR5, is able to provoke the production of various innate immunity-related components, including chemokines, inflammatory cytokines, and antimicrobial peptides. This treatment, in association with an antibiotic administration, induces synergistic antibacterial effects against infections caused by *S. pneumoniae* in mice. Innovative formulations targeting innate immune cells are also of great interest to improve vaccine efficacy. Matthijs et al. compare the immunogenicity of novel *M. hyopneumoniae* bacterin formulations associated with a cocktail of TLR1/2, TLR7, and TLR9 ligands in pigs. In this original paper, authors adapt the human-based approach of “blood transcriptional modules” to identify early immune signatures in the blood related to adaptive responses in pigs (2).

Traditionally, anti-infectious vaccines aim at targeting specific microbes by generating potent and long-lasting antigen-specific adaptive B and T cell immune responses. However, a growing body of evidence demonstrates that some vaccines can exhibit non-specific beneficial effects against heterologous infections. Cauchi et al. review the ability of a live attenuated pertussis vaccine to protect mice against heterologous airway infections, such as those caused by other *Bordetella* species, likely due the generation of cross-reactive B or (regulatory) T cells. The vaccine is also efficient against unrelated pathogens (IAV, RSV) and non-infectious inflammatory diseases (allergic asthma, contact dermatitis) and the authors discuss the presumed mechanisms involved in such protection, including trained innate immunity, as well as possible mechanisms underlying the anti-inflammatory effect of the pertussis vaccine.

In summary, the compilation of articles published within this Research Topic should give an overview of different innovative preventive and therapeutic approaches to fight against respiratory pathogens, including the rationale design of vaccine antigens and delivery/adjuvant systems in association with the understanding of immune mechanisms which contribute to vaccine efficacy, drug repurposing, and peptide therapeutics.

## Author Contributions

CCa, DD, and CCh contributed to the preparation, review, and revision of the manuscript. MC participated in the preparation of the Research Topic.

### Conflict of Interest

The authors declare that the research was conducted in the absence of any commercial or financial relationships that could be construed as a potential conflict of interest.
